# COVID-19 Vaccination and Gaps in India

**DOI:** 10.7759/cureus.37490

**Published:** 2023-04-12

**Authors:** Rajkamal Choudhary, Raj Rani Choudhary, Anjum Pervez

**Affiliations:** 1 Department of Medicine, Jawaharlal Nehru Medical College, Bhagalpur, IND; 2 Department of Obstetrics and Gynaecology, Nalanda Medical College, Patna, IND; 3 Department of Dialysis, Jawahar Lal Nehru Medical College, Bhagalpur, IND

**Keywords:** coverage, health, india, gaps, vaccination, covid-19

## Abstract

Introduction: The important variables that influence how many people are vaccinated against coronavirus disease (COVID-19) in India include vaccine skepticism, socioeconomic status, and multi-dimensional deprivation. Our preliminary research suggests that uncertainty about the safety of the COVID-19 vaccine has a large and detrimental effect on immunization rates.

Materials and methods: The Crucial Subsets Survey (CSS) is performed daily on Facebook to recruit participants for cross-section surveys by academic institutions, the Delphi Research Center, and the University of Maryland's Joint Program in Survey Methodology. Facebook will notify a portion of its daily users to take a vote. CSS adds behavior, attitude toward policy and preventive measures, economic consequences, and critical indicators to official reporting data.

Results: It has been estimated that a 30% drop in vaccination coverage may be attributed to a 1% rise in vaccine skepticism. Similarly, higher rates of multidimensional poverty are associated with lower rates of COVID-19 vaccine coverage. When the multidimensional poverty index (MPI), or the percentage of persons living in extreme poverty, rises by one unit, immunization rates fall by around half. It suggests that higher rates of socioeconomic hardship have unfavorable effects on health outcomes like vaccination rates. We also showed that gender is a major factor in influencing how internet availability affects vaccination rates and hesitation. We discovered that male vaccination rates went up in tandem with male internet use. This might be because of the digital divide and India's reliance on digital technologies like the COVID Vaccine Intelligence Network (COWIN), AAROGYA SETU, and Imphal, India, to assign and register for COVID-19 vaccinations, while males have greater digital excess than females. While male internet access is significantly and positively correlated with coverage, female internet access is significantly and negatively correlated with coverage. Women are less likely to seek medical care and have more vaccination reluctance than men, both of which contribute to this trend.

Conclusion: The government's strategy for disseminating information about the COVID-19 vaccination should prioritize reaching out to women. In order to recruit more women to vaccination clinics, it is important to raise public awareness about the need for immunization among women via the media and community outreach.

## Introduction

Despite vaccinating over a billion people against COVID-19, India has a gender gap. Studies show over 100 million Indians, mostly women, skipped the second COVID-19 vaccine dose [1]. This discrepancy casts doubt on India's "gender-neutral" COVID-19 vaccine information [1]. COVID-19 vaccination and awareness efforts did not target women.

India makes 60% of vaccines. Although distribution inequalities are more likely to cause problems than a shortage, the government wants to inoculate 300 million priority groups in the first phase. India's 1.35 billion people are 65.5% rural, according to the World Bank. This vaccine campaign should include rural residents, especially forgotten ones, and society's biases, not COVID-19. CO-WIN, a vaccine monitoring and registration software, organizes, distributes, and monitors India's massive COVID-19 immunization drive [2]. Aarogya Setu, India's COVID-19 contact tracking app, lets users schedule vaccinations. Aarogya Setu's CO-WIN interface lets users track COVID-19 exposure through contact tracking, self-assessment, and a QR-coded immunization certificate. Remote villages without internet will make vaccine distribution difficult. Nielsen found 36% of Indians were online in 2019. Worse, 70% of rural Americans have no internet. West Bengal, Bihar, Jharkhand, and Odisha have the lowest internet penetration.

The Indian government's digital information strategy supported its free and low-cost COVID-19 vaccination program [3]. The Indian government created the Aarogya Setu app and CoWin platform for online basic healthcare. Public health centers offer free vaccines, while private clinics charge. Vaccination registration was time-consuming and computer-savvy. The vaccine campaign excluded those without Aadhar cards, driver's licenses, or passports. The digital gap, which disadvantaged women and minorities, widened. The pandemic in India and elsewhere has affected women more than men, widening the workplace gender gap. Women dominate pandemic-fighting health and social workers. Women in India may be more susceptible to exposure to the virus because they spend more time caring for infants, the elderly, and sick family members, and because masks and other personal protective equipment are frequently made and sized for males [4].

The WHO-inspired Indian National Risk Communication Plan ignores local insights and culture. The Ministry of Health and Family Welfare created a Vaccine Communication document explaining the COVID-19 immunization campaign to reduce vaccine "hesitancy" due to safety and efficacy concerns. Social media influencers, community organizers, and frontline workers will boost COVID-19 vaccine support and public opinion [5]. A National Media Rapid Response Cell monitors traditional and digital media and responds quickly. Surprisingly, the National Media Rapid Response Cell values social media, influencers, and media monitoring. The strategy's digital and social media focus excludes women from COVID-19 discussions. Planning ignored women. No women. No print, radio, or TV campaigns have raised awareness of the disease's risk to pregnant women [6].

COVID-19 vaccinations have a gender gap. Only 143 million vaccines were given to women, compared to 167 million given to men. This gender gap is worrying, as the gender disparity in vaccination is far greater than 6%. The Co-WIN dashboard shows the total number of males and females immunized without gender breakdown. Policymakers are concerned because NITI Aayog policy think tank member V K Pal suggested more female patients be vaccinated to address the "skew in gender" issue [7].

Geography and poverty affect vaccine hesitancy. High-income countries immunize more than low- and middle-income ones. These societies value men over women. Men got COVID-19 vaccines first when supplies were low or payment was required. Due to side effects, homemakers may delay vaccination. Immunization rates are lower in countries where men make health decisions. Since most women choose their own healthcare, Tamil Nadu, Madhya Pradesh, and West Bengal have narrowed their vaccination gaps. Himachal Pradesh has the highest female immunization rate. Worker and retiree gender gaps were highest. Gender norms persist. Class and caste affect vaccination rates. Everyone should get the COVID-19 vaccine [8].

## Materials and methods

We have access to a real-time dataset revealing the total number of patients vaccinated throughout Indian states against COVID-19 infection, as reported by the Co-Win dashboard and the Indian Ministry of Health and Family Welfare. It is possible to gauge the degree to which people are reluctant to be vaccinated by looking at the results of the COVID-19 Consumption Survey.

Every day, the Crucial Subsets Survey (CSS) was conducted on Facebook as a recruiting medium for cross-section surveys by academic institutions, the Delphi Research Center, and the University of Maryland's Joint Program in Survey Methodology. A subset of daily Facebook users was invited to participate in a poll through a notice at the top of their News Feed. The survey includes standard questions on health, symptoms, diagnostics, COVID-19 vaccinations, and vaccination practices. The same, developed by experts in public health and surveys, has been approved by the Institutional Review Boards with the ethical number IRB/JNMC/11/123.

The large sample size of the CSS survey allowed us to use its findings to look into vaccine skepticism on a national and regional scale. It takes into account a statistically significant sample size of Facebook's enormous user base in India (over 320 million individuals). To ensure a broad geographic scope, we use a stratified random selection methodology within Indian states. The survey employs a two-stage weighting method to account for differences in Facebook users aged 18 and above within the sample frame and the whole Indian population, therefore eliminating any potential for error due to sampling. First, inverse propensity score weighting is used to make the Facebook user's sample more representative of the whole by adjusting for non-response bias.

A user's self-reported age, gender, and regional variables, as well as other characteristics that have been demonstrated to correlate with survey results in the past, are examples of the kinds of covariates that may be obtained from Facebook's own data. Second, Facebook uses post-stratification to ensure that its user base is evenly distributed across age and gender in each state, based on data from the United Nations demographic projections for 2020. Using the weights that are provided as part of the microdata, we can make sure that our survey population is representative of the general population and accounts for any differences between Facebook users and the rest of the population, as well as a Facebook user's propensity to take the survey in the first place. We were familiar with the limitations inherent in CSS layout. Our use of the data was limited by the following considerations, which we are aware of: look for regions with noticeably varied degrees of hesitancy by comparing them to others across the globe in order to track changes over time, such as growing or dropping vaccination positivity and varying vaccine skepticism among Indian states.

Research highlighting the benefits of the CSS was included in a systematic review on the validation of web-based surveys, and it showed that these types of large online surveys like the CSS provide continuous, real-time indicators of important outcomes that are not subject to public health reporting delays and backlogs. The CSS is a useful supplement to official reporting data because it gives information on behavior, attitudes toward policy and preventive measures, economic implications, and critical indicators that are not normally documented in public health surveillance systems.

In order to calculate the extent of vaccine skepticism in India, we analyzed the results of the CSS from the beginning of 2020 all the way to the end of 2022. The vaccination hesitation is mapped by asking respondents if they would be vaccinated against COVID-19 if they were granted the option. The respondents were given the following choices for their responses: without a doubt; perhaps; probably not; definitely not. In order to look at how the use and interest in the COVID-19 vaccination have changed over time, we pooled the data by month. Repeat cross-sectional surveys were used to the data to obtain estimates. The NITI AAYOG report is the source of information on economic hardship or the multi-dimensional poverty count. Estimates for regular immunization, years of education, and internet connection for men and women at the state level were captured using survey data from the National Family Health Survey Round 5.

The model

First, we used daily vaccination data from the Co-Win dashboard and an ordinary least squares regression model to investigate what variables determine coverage and gaps in the COVID-19 vaccine across Indian states. We have used both linear and log regression models to examine whether or not there is a correlation between the rate of vaccine wastage, the percentage of the population that falls into the "multi-dimensionally poor" category, the percentage of the population that is hesitant to get vaccinated against COVID-19, and internet access.

Dependent variable

Our study relies on the COVID-19 vaccination coverage, or the percentage of the population that has received all recommended doses of vaccine against this disease.

Independent variable

Our research considers both demand and supply variables that may have an effect on rates of COVID-19 immunization. We have utilized data from the COVID-19 consumption survey, specifically the amount of vaccination hesitancy (the percentage of the population unwilling to get the vaccine). Those who are more skeptical of the COVID-19 vaccine tend to have lower immunization rates. To further investigate the factors that influence whether or not people get the COVID-19 vaccination, we have additionally examined many socioeconomic indicators. We have utilized the National Institution for Transforming India (NITI) Aayog's multidimensional poverty indicator to cover the supply side. When creating an indicator of socioeconomic disadvantage, the Multi-Purpose Indicator (MPI) considered health, education, and quality of life in all Indian states to be equally significant factors as the others. Some examples of indicators that fall under these umbrellas are malnutrition, infant mortality, years of schooling, access to cooking fuel, sanitation, drinking water, electricity, housing, assets, financial inclusion, and so on.

It stands to reason that healthcare and vaccination results would be worse in states with higher MPI ratings or a larger proportion of people living in multidimensional poverty. The impact of education and technology on COVID-19 vaccination rates is shown using literacy and internet access with other socioeconomic indicators as independent variables. Both might provide insight into the question of why there are discernible variations in vaccination rates across geographic regions. A person's likelihood of being vaccinated may increase, for example, if they can easily and quickly register for vaccines, get immunization certificates, etc., online. However, even among people who have access to the internet, exposure to misleading news and common misunderstandings regarding COVID-19 immunizations online may dissuade persons from becoming vaccinated. Vaccination rates may rise if the former predominates, and vice versa. Awareness of healthcare options and the COVID-19 infection may also be affected by educational factors, which in turn may affect vaccination decisions.

Then, we used the Loess regression model to look at the factors that could have an effect on COVID-19 vaccination reluctance separately. As separate regressors, we have employed factors like education level, computer ownership, and the multidimensional poverty index. This model was created with the intention of making fewer assumptions about the relationship between the response variable and the predictor factors. Simply stated, we use locally weighted fits to ascertain the significance of the association between the response and the predictor variables. The variance of residuals and the predictor error term are often reduced in locally weighted fits. Using a locally weighted regression function, one may get a close approximation of the curve's form, which is determined only by the data. It enables the data to determine the shape of the connection between the answer and the predictor variables, rather than assuming any particular shape.

## Results

We evaluated two different models, linear and log, within the demand and supply framework of vaccine uptake to determine if vaccine anxiety, internet access, and multi-dimensional deprivations have a significant role in determining COVID-19 vaccination uptake or coverage. A major decrease in COVID-19 immunization rates is first seen, which we attribute to vaccine hesitancy (p = 0.01). The gap between the fully vaccinated proportion and the percentage shift in vaccine resistance is rather considerable. Increases in vaccine skepticism are associated with a 30% reduction in vaccination coverage (model 2). Second, we have included measures of personal disadvantage (MPI) as an independent regressor in our analyses. To no one's surprise, we discovered a statistically significant inverse relationship between the MPI and the share of the population that has received all recommended vaccinations across states (p 0.05). When the MPI (or the percentage of people living in extreme poverty) increases by one unit, immunization rates drop by half.

It implies that higher rates of socioeconomic hardship or the prevalence of people living in multidimensional poverty have a negative impact on health outcomes like vaccination rates. As a result of financial constraints, people in extreme poverty are less likely to prioritize their health, particularly preventative care options like vaccinations. Vaccination costs money, and they cannot afford to miss workdays to pay for it. For all these reasons, vaccination rates are lower in low-income communities. Third, there is a strong correlation between having internet access and being male but a negative correlation between having internet access and being feminine. This finding suggests that rising rates of vaccination coincide with rising rates of male internet access. It is true that males are more likely to have internet access than females (coefficient = 0.6, p = 0.01). Vaccination rates rise by 60% for every 10% increase in the percentage of men who have access to the Internet. While more and more women are gaining access to the internet, fewer children are being vaccinated. The coefficient for women's internet access is -0.53 (p = 0.01). Similarly, a 50% drop in vaccination coverage is shown whenever there is an increase in the number of women who have access to the Internet.

Determinants of COVID-19 vaccine coverage in India

Anti-Vaccination Feelings Are Fitted by Scatter Plots with Bivariate Regression

**Figure 1:**
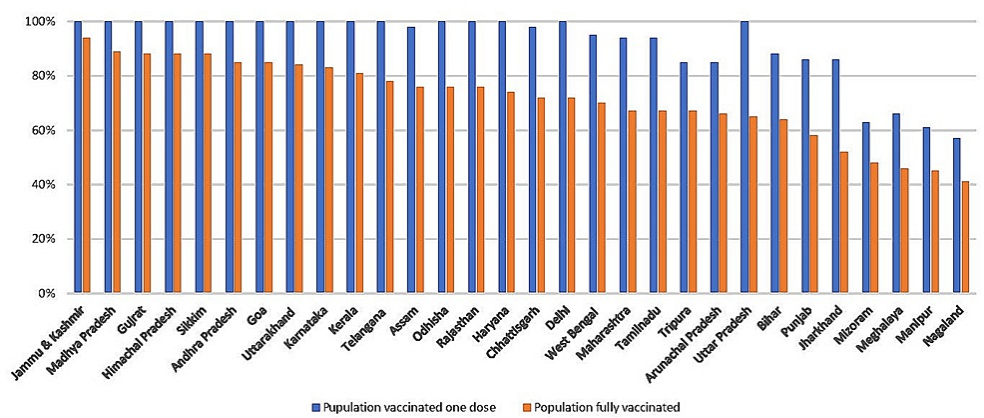
Progress of COVID-19 vaccination in India

Since the beginning of the vaccine campaign, India has vaccinated an average of 14 million people every month against COVID-19. Only a few states have regularly reached vaccination rates of 90% or more among their eligible population, whereas the vast majority have not even reached the 60% to 70% coverage level in the one-year duration time shown in Figure 1.

According to our model, vaccination skepticism is a possible contributor to lagging states' poor coverage. A 30% drop in vaccination rates has been linked to a 1% rise in vaccine skepticism.

We analyzed the results of the CSS survey conducted between January and December 2021 to gain a comprehensive picture of COVID-19 vaccine hesitancy and to comprehend the patterns of reluctance across states. We discovered that acceptance rates for the COVID-19 vaccination in India range from 53% to 95% throughout the country's several states. According to another study in India that used the same CSS dataset, it was predicted that around 45% of those polled would choose to get COVID-19 vaccinations on a national basis. Yet a sizable minority (29%) indicated reservations about getting the immunization. The top three reasons people were afraid to get the vaccination were as follows: worry about possible adverse effects, fear of what other people would think, and a desire to wait and see whether the vaccine is safe. One of the most recent research projects utilized CSS data to show that parents who sent their kids to school in person had a higher chance of experiencing complications from the COVID-19 virus.

Another study based on the CSS survey showed that there are significant regional and worldwide gaps between the sexes in key health and non-health parameters. Using data from the CSS survey, this study revealed that vaccination reluctance was substantially greater among women (25.6%) than among males (22.3%). As time went on, the gender gap shrank, and by September 2021, it had been effectively filled on a worldwide scale. Women are more likely to be apprehensive about getting the COVID-19 vaccination, according to research that used the CSS survey to investigate the variables related to vaccine reluctance in the United States. Increased hesitation was associated with both younger age and non-Asian ethnicity.

Our research shows that COVID-19 vaccine reluctance is particularly significant in the Indian states that have had the most difficulty increasing their immunization coverage. Examples of states with poor vaccination rates include Tamil Nadu, Punjab, Nagaland, Meghalaya, Mizoram, Manipur, Jharkhand, etc. [9]. Although vaccination hesitancy is minimal and coverage is strong in several states like Himachal Pradesh, Sikkim, Goa, Madhya Pradesh, Kerala, Uttarakhand, etc., using a linear regression model, we investigated whether or not vaccine apprehension (as indicated by the CSS survey) was a contributing factor to low COVID-19 vaccination coverage (the percentage of the population that has received all recommended vaccinations). While other factors were taken into account, we still identified a negative and statistically significant correlation between vaccine hesitation and overall immunization rates for COVID-19. As a result, overcoming vaccine hesitancy should be prioritized in order to increase immunization rates in lag states [10].

We investigated whether or not adults' openness to the use of vaccines like COVID-19 is influenced by the degree to which they accept regular child immunization. We used a regression model to determine that being vaccinated as a kid increased the probability that one would receive a vaccine against COVID-19. Based on the scatter plot and regression line, it seems that there is a positive correlation between the two variables. Our findings indicate that high rates of child immunization and coverage with the COVID-19 vaccine are prevalent in states like Himachal Pradesh, Jammu & Kashmir, Karnataka, Goa, Sikkim, Telangana, etc. Nonetheless, regular and COVID-19 vaccination coverage is poor in states like Jharkhand, Bihar, Meghalaya, Nagaland, Mizoram, Punjab, Manipur, etc.

Vaccine hesitancy factors and the vaccine gap in India

We looked at what variables, if any, would have an effect on an individual's reluctance to have the COVID-19 vaccine. The World Health Organization (WHO) claims that vaccination skepticism shifts over time and between locations and diseases. The World Health Organization's Strategic Advisory Group of Experts defines vaccine hesitancy as "a delay in acceptance or rejection of immunization despite the availability of vaccination services." It is noteworthy to note that vaccine skepticism varies in intensity from country to country, depending on the severity of their health and economic problems. Factors such as religious identification, gender, political ideology, and trust in medical and scientific institutions are connected to vaccination hesitation in general and COVID-19 vaccine skepticism in particular. It is still the case that there are racial and economic gaps in health that were exacerbated by the pandemic. Most at risk for getting COVID-19 are those from underrepresented groups, those with lower incomes, and those with less education (a bachelor's degree or less). Non-college-educated Americans have a 42% lower vaccination rate than college graduates. People with lower incomes and those who live in rural areas are less likely to be immunized.

**Figure 2:**
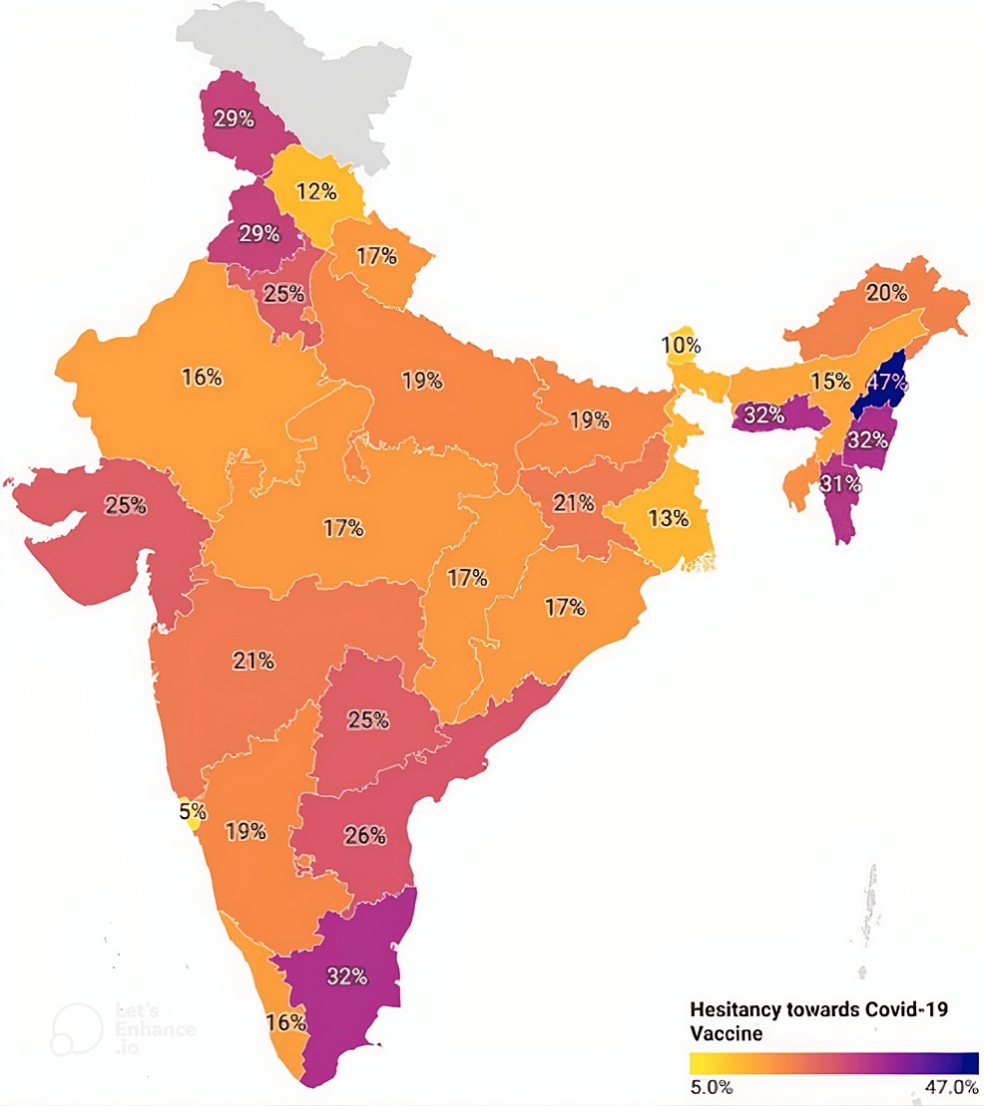
Trends of vaccine hesitancy in India

Concerns about COVID-19 vaccine safety, skepticism of new vaccinations, the speed at which new COVID-19 vaccines are developed and approved for use in humans, a lack of evidence about vaccine safety, rumors, and misinformation spread via social media all contribute to a general lack of faith in these products among the Indian public. Recently, concerns have been raised about the ability of vaccines to protect against emerging threats like Omicron. Fear of adverse reactions is another major factor contributing to vaccination reluctance. A minority of people (across faiths) thought that vaccinations were incompatible with their way of life. There is, therefore, an immediate need to comprehend the reasons that cause vaccination reluctance and how that affects people's openness to or rejection of COVID-19 vaccines. Vaccination hesitation rates in India's several states range from 47% in Nagaland to 5% in Goa, as shown in Figure 2.

This wide range of rates indicates that vaccine hesitancy is not evenly distributed throughout the country.

## Discussion

To begin, we examined the processes at work in India's COVID-19 vaccination rate and the extent to which they were affected by socioeconomic factors. Using a new regression model, we investigated the factors that may have an independent influence on vaccination hesitancy against COVID-19. We discovered that states with low vaccination coverage rates also had significant levels of vaccine reluctance for the COVID-19 strain. Our findings indicate that a 1% rise in vaccine skepticism is associated with a 30% decrease in vaccination coverage. So, COVID-19 vaccine skepticism might be a contributing factor to poor immunization rates in certain delayed states. This finding suggests that efforts to achieve universal immunization against COVID-19 should concentrate on reducing vaccine skepticism. Second, we checked to see whether lower COVID-19 immunization rates are related to socioeconomic inequalities across states as assessed by the MPI. Extreme poverty is prevalent in several Indian states. These include Uttar Pradesh, Bihar, Jharkhand, Madhya Pradesh, Chhattisgarh, Assam, Meghalaya, Rajasthan, Odisha, and many more (MPI). When compared, these states, like Goa, Jammu and Kashmir, Andhra Pradesh, Rajasthan, etc., have the fastest levels of poverty reduction [9]. During the past few decades, states that have invested heavily in health and education have consistently outperformed those that have not in terms of indicators like gross domestic product (GDP) growth, infant mortality, access to prenatal care, access to clean water and electricity, access to decent housing, the availability of medical facilities, and other economic assets [9].

However, since they invested in a strong healthcare infrastructure, they have been able to efficiently handle critical health situations like COVID-19. Using a multi-dimensional poverty indicator, we discovered a negative connection between socioeconomic status and COVID-19 immunization rates. Reducing multi-dimensional poverty has far-reaching effects on people's lives, including changes in their attitudes and behaviors about health, education, illness prevention, and more [10]. Generally speaking, individuals invest more in their health and their education as their economic situation improves. Further, when health education and awareness increase, states' health outcomes as a whole, including health infrastructure, improve, which has beneficial effects for handling crises like COVID-19 and expanding access to vaccinations [10,11].

The allocation and registration of COVID-19 vaccinations were handled by COWIN and AROGYA SETU. When compared to women, men had better access to the internet, which gives them an edge when it comes to things like registering for programs on time, finding nearby vaccination centers, downloading proof of immunization, and so on. To further illustrate the gender gap in COVID-19 vaccination uptake, out of a total of 1.7 billion doses given in India, only 143 million were given the vaccine to women as compared to 167 million given to men. Gender differences in COVID-19 immunization rates suggest that more men than women are receiving the vaccine, a trend that is reflected in the numbers. Vaccination rates are rising across the board, but any improvement in male access to the COVID-19 vaccine would have a disproportionately big effect on absolute coverage since more men are becoming vaccinated. On the other hand, there is a statistically significant inverse correlation between female internet access and vaccination rates. Because of vaccination reluctance, especially among women, this has occurred [12]. Multiple studies by groups like the Infrastructure Development Finance Company Limited (IDFC) Institute in Mumbai, the National Council of Applied Economic Research (NCAER), etc., found that females are more likely to be wary about COVID-19 vaccinations than men because of misinformation disseminated on social media [13]. Women are more likely to believe rumors and false information about the dangers of immunizations, infertility, and menstruation. Women are far less likely to make use of healthcare resources and family time than men are. Neglect of women's health in India stems from systemic issues such as unequal access to medical treatment and the unequal distribution of family resources between the sexes. There is a widespread belief that women should take less care of their health than men since they stay home and care for their families while men go out to work [14].

Existing studies in India and elsewhere corroborate our findings. About half of Indians (58%) are certain to be vaccinated, according to a population-based longitudinal study performed on 3000 individuals across four Indian states in June 2021 to map COVID-19 vaccine acceptability, hesitance, and resistance. The poll classified vaccination reluctance into two groups: those with low reluctance (28%) who are likely to be vaccinated, and those with strong reluctance (7%) who would not. Moreover, the poll found that demographic factors including age, gender, location, level of education, and socioeconomic position all play a role in vaccination skepticism. The poll found that women were more reluctant to be vaccinated than men were, and that those with a master's degree or above were more likely to support vaccination than those with fewer than 10 years of education. People in higher income brackets were more likely to have been vaccinated than those in lower income brackets. In high-COVID-19 burden nations, such as France, Germany, and Sweden, women are more likely to accept a vaccination than males, according to a cross-country investigation analyzing the link between socio-economic characteristics such as age, gender, and education with vaccine uptake. In Canada, Poland, France, Germany, and the United Kingdom, people over the age of 50 are more willing to obtain immunizations than individuals under the age of 50. Similarly, the vaccination is expected to be accepted by educated people in India, Ecuador, the United States, and Germany. In Canada, Spain, and the United Kingdom, however, greater levels of education are correlated with lower vaccination rates [11-13].

We dug further to discover what factors could explain people's reluctance to obtain the COVID-19 vaccine. We hypothesized that people with greater levels of literacy would be healthier because they would be more aware of health issues and more likely to take preventative measures like being vaccinated. We observed that vaccination apprehension is inversely proportional to literacy. States with a low literacy rate have a higher rate of vaccination skepticism. Unexpectedly, our findings reveal that first-time vaccination reluctance rises as literacy levels rise. Nonetheless, as more people in society are able to read and write, vaccination skepticism goes down. Policymakers should take note of the finding that vaccine awareness efforts are effective until a critical mass of the public can read and comprehend information on the benefits of health programs like vaccination. This suggests that efforts to reduce vaccination skepticism should be tailored to the specific areas in question and should take into account the overall degree of education in those places. Furthermore, we showed that exposure to digital technologies like the Internet is a significant factor in determining whether or not people are open to receiving COVID-19 vaccinations. Our findings show that easy access to digital technology has both positive and negative consequences. Timely vaccination registration, finding local vaccination clinics, retrieving digital vaccination certificates, etc., all benefit from an Internet connection. Patient refusal to receive the COVID-19 immunization may be due to widespread misinformation and misunderstandings. Machingaidze and Wiysonge [15] hypothesized that the proliferation of digital technologies like the Internet and smartphones will lead to a rise in internet and social media usage in low- and middle-income countries; our results corroborate this prediction. While this presents a number of challenges, including rumors, disinformation, anti-vax attitudes, fake news, and a lack of information about vaccinations, it is also a fantastic tool for self-education, awareness, access to vaccines, etc., all of which are essential elements of vaccination decision-making [15].

Our study's empirical findings may inform the development and refinement of policy responses to COVID-19. In several Indian states, a sizeable minority of the population still refuses vaccinations. There is a strong correlation between the average number of years spent in education and the availability of digital technologies like the internet, both of which have a bearing on vaccination skepticism. Vaccine skepticism is on the rise in many states due to the proliferation of false information online, so it is important to implement measures that might curb this trend. It is possible to launch COVID-19 vaccination awareness campaigns in regions with a low-literate population. Long-term expenditures in healthcare infrastructure and socioeconomic indices are linked to increased use of healthcare services, such as vaccinations. States that have invested much in healthcare and education over the last few decades have well-developed healthcare infrastructure and are better prepared to handle sudden health catastrophes like COVID-19 [16].

As a result of the COVID-19 pandemic, the country's healthcare system is at breaking point. The myth that city hospitals can handle emergency situations has been completely debunked. This is especially crucial in rural regions, where access to healthcare is limited. Subsequent COVID-19 waves have shown the need to further strengthen healthcare delivery in both metropolitan and rural locations. When dealing with medical crises like the one caused by the COVID-19 virus, the healthcare system plays a critical role in preventing further harm. States with more developed healthcare systems will be better able to deal with the COVID-19 pandemic and inoculate residents more quickly [15]. After accounting for confounding factors, we observed that improvements in healthcare infrastructure were associated with higher rates of COVID-19 immunization. Thus, as state healthcare systems improve, more people will get vaccinated against COVID-19.

## Conclusions

The report suggests that vaccine skepticism, poverty, poor health facilities, low education, and limited internet access lower COVID-19 immunization rates. COVID-19 vaccination rates were higher in states with lower vaccine hesitancy, poverty, health infrastructure, literacy, etc. COVID-19 immunization rates were low in poor, vaccine-skeptical, and underserved areas. As the world and research advance, countries need a data management and knowledge-sharing system. Governments should share vaccines since no one is safe until everyone is safe. Indian culture should guide decisions. The COVID-19 vaccination should target women. Media and community outreach help women get vaccinated.
